# Expression profile of the *Plasmodium falciparum* intra-erythrocytic stage protein, PF3D7_1363700

**DOI:** 10.1186/1475-2875-12-66

**Published:** 2013-02-19

**Authors:** Renee N Roberts, Maggie S Schlarman, Michael M Kariuki, Alexis N LaCrue, Ruguang Ou, Brenda T Beerntsen

**Affiliations:** 1 Department of Veterinary Pathobiology, University of Missouri, Columbia, MO, USA; 2 Molecular Microbiology and Immunology and Veterinary Pathobiology Joint Graduate Program, University of Missouri, Columbia, MO, USA; 3 Department of Global Health, University of South Florida, Tampa, FL, USA

**Keywords:** *Plasmodium falciparum*, PF3D7_1363700, Intra-erythrocytic stage, Gene expression, Protein expression

## Abstract

**Background:**

Efforts to control malaria are demanding due to drug-resistant parasites, insecticide-resistant mosquitoes and poor health infrastructure in malaria-endemic countries. Therefore, the research and development of additional malaria control methods are crucial. For host-parasite interactions, surface antigens and secreted proteins are likely to be involved in infectivity and invasion of host tissues and therefore can be effective targets for control by vaccines, drug therapy, or novel mosquito control methods. In an effort to identify and characterize genes that may have a role in host-parasite interaction, this study describes the expression profile of *Plasmodium falciparum* PF3D7_1363700.

**Methods:**

A *P. falciparum* gene, *PF3D7_1363700*, was identified by a search of the annotated *Plasmodium* genome database. Protein alignments of PF3D7_1363700 orthologues from various *Plasmodium* species were performed to demonstrate protein similarity. Transcript expression profiles of PF3D7_1363700 were determined via reverse-transcriptase PCR and protein expression was investigated by immunofluorescence assays, western blot analysis and green fluorescent trafficking studies.

**Results:**

The PF3D7_1363700 protein demonstrates significant similarity with orthologues in other *Plasmodium* species and appears to be unique to Apicomplexans. The *PF3D7_1363700* transcription profile demonstrated expression during the intra-erythrocytic, oocyst sporozoite, and salivary gland sporozoite stages while the PF3D7_1363700 protein was only detected during the intra-erythrocytic stages.

**Conclusions:**

This research utilized an *in silico* approach to identify a well-conserved protein known as PF3D7_1363700. By molecular, biochemical and cellular analyses, PF3D7_1363700 was discovered to be an intra-erythrocytic-specific stage protein that is unique to Apicomplexans.

## Background

Malaria is a mosquito-borne disease responsible for approximately 216 million human infections and an estimated 655,000 deaths annually, with most deaths occurring in African children [1]. Novel therapeutic intervention methods and other ways to complement or replace existing malaria control methods are a necessity to combat malaria especially when drug-resistant parasites and mosquitoes resistant to commonly used insecticides are a continuing issue and there is no effective malaria vaccine available [2-5]. New bio-informatics analyses of the *Plasmodium* genome and proteome have provided opportunities to identify and search for protein candidates that potentially can serve as effective targets for vaccines, drug therapy, and/or novel mosquito control methods.

In an effort to discover new anti-malarial targets, data mining of the *Plasmodium* genome database (PlasmoDB; [6]) was performed to specifically select *Plasmodium* proteins expressed on the surface of the malaria parasite, which are often ideal targets. As a result, a novel *Plasmodium falciparum* gene, *PF3D7_1363700,* also previously known as *PfMAL13P1.319,* was identified as a 2.3-kb gene that encodes a 790-amino acid protein. Although initial data mining of PlasmoDB identified PF3D7_1363700 as a sporozoite surface protein via protein mass spectrometry, the research described herein analyzed PF3D7_1363700 via molecular, biochemical and cellular assays and discovered that it is likely a secreted protein expressed during the late intra-erythrocytic stages. The PF3D7_1363700 protein has orthologues in various *Plasmodium* species and appears to be unique to Apicomplexans since no orthologues of PF3D7_1363700 were identified in higher eukaryotic organisms. Here, this research investigated the transcript and protein expression profile of PF3D7_1363700 in an effort to understand the potential role of PF3D7_1363700 in malaria parasite biology.

## Methods

### Mosquito and *Plasmodium* parasite maintenance and transmission

*Anopheles stephensi* were reared as previously described [7,8] and used for maintaining the *P. falciparum* life cycle and parasite transmission. The *P. falciparum* NF54 strain and 3D7HT-GFP parasites (a constitutively green fluorescent protein (GFP)-expressing parasite line) were maintained according to a standard protocol [8,9] and used for mosquito-parasite infections. Mosquito parasite transmission studies also were performed according to standard protocol [8]. Gametocyte cultures used for mosquito parasite transmission and for transcript and protein analysis were established by maintaining a 0.5% parasitaemia NF54 culture supplemented with 10% human serum (A+ male, Interstate Blood Bank) at a 3% haematocrit and changing medium every other day for 16 days [10,11].

Enrichment of intra-erythrocytic stages was performed by synchronizing parasites using 5% D-sorbitol (Sigma) according to standard procedure [10]. Briefly, parasite-infected cells were pelleted at 2,000 × g for 5 minutes and resuspended with five pellet volumes of 5% D-sorbitol for 5 minutes at room temperature. Infected RBCs were centrifuged again at 2,000 × g for 5 minutes and washed three times with 10 pellet volumes of RPMI 1640. After removal of the final wash, parasite-infected RBCs were resuspended in culture medium, gassed with a 3% O_2_, 3% CO_2_ and 94% N_2_ gas mixture and incubated at 37°C.

All experiments described herein were approved by and performed in accordance with the Institutional Biosafety Committee (IBC), the Institutional Review Board (IRB), and the University of Missouri Institutional Animal Care and Use Committee (IACUC).

### *Plasmodium falciparum* parasite isolation

#### Sporozoite isolation

To obtain *P. falciparum* oocyst and salivary gland sporozoite stages, 3–7 day old mosquitoes maintained using conditions reported previously were exposed to an infected bloodmeal and dissected 8–10 days post-exposure (PE) for *P. falciparum* oocyst sporozoites and 14–21 days PE for *P. falciparum* salivary gland sporozoites. Isolation of oocyst sporozoites was performed by removing the mosquito midgut, transferring it to a slide with a drop of 1X PBS (137 mM NaCl, 2.7 mM KCl, 10 mM Na_2_HPO_4_, and 2 mM KH_2_PO_4_), then rupturing the midgut oocysts by placing a coverslip onto the midgut to release mature sporozoites from the oocysts. *Plasmodium* salivary gland sporozoites were isolated by dissecting the infected salivary glands and placing them in a tube of 50 μl 1X PBS where sporozoites were released from the salivary glands by vigorous pipetting and processed as previously described [7].

### Axenic liver stage isolation

*Plasmodium falciparum* axenic liver stage parasites were obtained in the absence of host hepatocytes as previously described [8,12].

### Obtaining parasite lysates for DNA and protein analysis

To obtain oocyst and salivary gland sporozoite lysates, sporozoites isolated from oocysts (n = ~100 mosquitoes) or salivary glands (n=~100 mosquitoes) were either homogenized and used for RNA isolation or resuspended with 1X reducing buffer (0.5% bromophenol blue, 4% SDS, 10% 2-β-mercaptoethanol) for protein analysis. In order to prepare erythrocytic stage parasite lysates (i.e., a mixture of rings, trophozoites, schizonts and gametocytes), infected blood was centrifuged at 2,700 × g for 5 minutes and the supernatant discarded. The pellet was resuspended in a solution containing 0.05% (w/v) saponin (Sigma) in RPMI 1640 medium and incubated for 2 minutes at room temperature in order to lyse the RBCs and release parasites. Saponin-treated cells were pelleted at 2,700 × g for 10 minutes and washed at least twice with RPMI 1640 medium. The resulting pellet either was resuspended in 1X PBS for genomic DNA isolation (Qiagen DNeasy Blood and Tissue Kit), 1X reducing buffer for protein analysis or TRIzol reagent (Invitrogen) for RNA isolation.

### Identification and sequence analysis of PF3D7_1363700

Initial efforts to identify novel sporozoite surface or secreted proteins that may be involved in sporozoite invasion of host tissues were performed by data mining PlasmoDB [6]. The data-mining search specifically aimed to select for uncharacterized genes that expressed protein during the sporozoite stage, although future analyses suggested otherwise, and was predicted to be a surface or secreted protein with a signal peptide. The PF3D7_1363700 protein was identified and protein sequences were subjected to BLAST analysis within PlasmoDB [6] and National Center for Biotechnology Information (NCBI) [13] databases to determine protein similarity. Additional analyses were obtained using Expert Protein Analysis System (ExPASy) proteomic tools such as TargetP and SignalP for signal peptide/anchor predictions, PSORTII, TMAP, and DGPI for localization, transmembrane and GPI anchor predictions, and Pfam and Prosite for classifying family proteins and identifying functional sites, and other patterns/profiles [14].

### Reverse-transcription PCR (RT-PCR) and PCR analysis

To examine the expression of PF3D7_1363700 in oocyst sporozoites, salivary gland sporozoites, axenic liver stages and intra-erythrocytic stages, total RNA was isolated using the TRIzol reagent (Invitrogen) according to the manufacturer’s instructions. Total RNA was DNase treated (Ambion) to remove residual genomic DNA and 2–3 μg of RNA was used as a template for Superscript™ First-Strand Synthesis System Reverse Transcriptase-Polymerase Chain Reaction (RT-PCR; Invitrogen) to make first-strand cDNA. PCR amplification of the cDNA was performed with gene specific forward (F) and reverse (R) primers, *PF3D7_1363700*- F: 5^′^-GAGCACATCGATGGGGAGG-3^′^ and R: 5^′^-TTAACTTGAGTCTAGACCTCTGG-3^′^; *Plasmodium falciparum merozoite surface protein* (*PfMSP-1*) - F: 5^′^-AAACTAGAAGCTTTAGAAGATGCAG-3^′^ and R: 5^′^-CCCTTCTTCATTATCTGCATTCTT-3^′^; *Plasmodium falciparum sexual-stage antigen* (*Pfs230*) - F: 5^′^-TATAACGCTGAAGAATCTATTCCTC-3^′^ and R: 5^′^-GTACTAGGTTAAAGCTATCCGAGG-3^′^; *P. falciparum circumsporozoite* (*PfCS*) - F: 5^′^-CGCGGATCCATGATGAGAAAATTAGCTAT-3^′^ and R: 5^′^-CCGCTCGAGCTAATTAAGGAACAAGAAG-3^′^ and *P. falciparum heat shock protein 70* (*PfHSP70*) - F: 5^′^-AGGTATAGAAACTGTGGGTGG-3^′^ and R: 5^′^-GATTGGTTGGCATACAGCTTC-3^′^ control gene primers were used to detect asexual blood stage, gametocyte, sporozoite, and liver stage transcripts, respectively. *Plasmodium falciparum* NF54 genomic DNA was used as a positive control template and a no reverse-transcriptase cDNA was utilized to control for genomic DNA contamination.

PCR amplifications were carried out using the following conditions: 1.5 mM MgCl_2_, 0.2 mM dNTP, 2.5 U Taq polymerase (Promega), 0.4 mM of each primer, and ~100 ng of cDNA template to a total reaction volume of 50 μl. PCR amplification was performed as follows: 1) initial denaturation at 94°C for 5 minutes; 2) denaturation at 94°C for 30 seconds; 3) annealing at 50°C for 45 seconds; 4) extension at 62°C for 2 minutes and 5) final extension at 62°C for 10 minutes. Each amplification cycle was repeated 34 times. Resulting products were separated via 1% agarose gel electrophoresis and stained with ethidium bromide for visualization.

### Generating anti-PF3D7_1363700 antibodies

To produce antibodies for immunocytochemistry studies, the signal peptide region was omitted and the remaining *PF3D7_1363700* gene was divided into thirds (i.e., fragments A-C) due to the relatively large size of the gene and difficulties when producing the entire protein. The N-terminal A fragment of PF3D7_1363700 (PF3D7_1363700-A) was chosen for commercial antibody production so that the truncated-protein produced from any gene-disrupted mutant parasites could be detected. First, PCR amplification was performed using gene specific forward and reverse primers for this region (*BamHI* and *NotI* restriction enzyme sites are italicized):

Fragment A: F: 5^′^-CG*GGATCC*TTGATCATAAAATATAATGTCAAG-3^′^ and R: 5^′^-AGT*GCGGCCGC*TTATCCTATTTCATGTAATAACAAA-3^′^. Then, this fragment was cloned into the pCR®II-TOPO® vector (Invitrogen), sequenced, double-digested and cloned into the pET-32a expression vector (Novagen). Recombinant DNA plasmids were transformed into Rosetti-gami cells (Novagen) and the cells were induced with 1 mM IPTG. Inclusion bodies and soluble fractions were obtained by sonicating harvested cells with 10-second bursts at a medium intensity setting using the Sonic Dismembrator (Fisher Scientific), freezing the lysate in liquid nitrogen for one minute, and thawing the lysate in a 42°C water bath. This rapid freeze thaw-sonication procedure was repeated at least three times. Removal of soluble protein was obtained by centrifugation at 25,000 × g for 20 minutes and the inclusion bodies were used for antibody production since PF3D7_1363700-A recombinant protein was present in inclusion bodies via Western blot analysis. Commercial rabbit polyclonal anti- PF3D7_1363700-A antibodies were produced by Sigma Genosys. The first immunization consisted of 200 μg of recombinant PF3D7_1363700-A mixed with Freund’s Complete Adjuvant while subsequent immunizations (i.e., a total of five injections) used 100 μg of PF3D7_1363700-A plus Incomplete Freund’s adjuvant. Recovered antibodies were titrated and tested for recombinant PF3D7_1363700-A detection. Pre-immune serum also was obtained before immunizations.

Polyclonal anti- PF3D7_1363700 antibodies and pre-immune serum were IgG-purified using the Melon Gel IgG Purification Kit (Pierce). To further purify antibodies to reduce cross-reaction against tissues and pET-32a bacterial protein lysate, two purification methods were performed for either Western blot analysis or immunofluorescent assays (IFA). For antibodies used in Western blot analyses, anti-PF3D7_1363700 antibodies were purified as previously described by Schlarman and colleagues [8]. Antibodies for IFA were purified using a pre-absorption method as previously described [7], which consisted of pre-absorbing the antibodies against protein lysates of mosquito midguts, mosquito salivary glands, human RBCs and pET-32a bacterial protein lysates. Briefly, protein lysates from mosquito midguts or salivary glands were made by homogenizing mosquito midguts (n = 10) or salivary glands (n = 20) in 1X PBS, respectively, while protein lysates from RBCs were obtained by saponin-treatment. All lysates were resuspended in reducing sample buffer, denatured at 95°C for 10 minutes and separated on a short, 10% SDS-PAGE mini-gel (approximately 2.5 cm). Proteins were transferred to nitrocellulose and each lane was cut into nitrocellulose strips and individually incubated with diluted anti-PF3D7_1363700 antibodies or pre-immune serum. Anti-PF3D7_1363700 antibodies and pre-immune sera were each incubated with three strips of proteins from midguts, salivary glands, RBCs, and pET-32a protein lysates.

### Western blot analysis

Erythrocytic stage parasite lysates (including mutant PF3D7_1363700-green fluorescent protein (GFP) lysates) were prepared by saponin-treating parasite-infected RBCs and resuspending the pellet with a 1:1 dilution of 1X reducing buffer and 1X PBS. Lysates were separated on a 10 or 12% SDS-PAGE gel and transferred onto a nitroceullose membrane. Membranes were blocked with 5% non-fat dry milk-Tris buffered saline (TBS; 0.15 M NaCl, 50 mM Tris) for approximately 1 hour at room temperature, washed three times in 1X TBS, then incubated with a 1:100 dilution of purified pre-immune serum (i.e., used a negative control to show no cross-reacting antibodies), rabbit-anti-PF3D7_1363700 antibodies, or anti-GFP in TBST (TBS/0.05% Tween 20) overnight at 4°C on a shaker. After overnight incubation, the membrane was washed three times with 1X TBST, and incubated with a 1:20,000 dilution of secondary antibody (i.e., peroxidase-conjugated anti-rabbit IgG or anti-mouse IgG; Sigma) and visualized using an enhanced chemiluminescence (ECL) detection system (Amersham Biosciences). There were three biological replicates performed for Western blot analyses.

### Immunofluorescence assay (IFA) and confocal microscopy

For immunofluorescence analysis of the PF3D7_1363700 protein during the intra-erythrocytic stages, parasite-infected blood smears (the same parasites were used for transcriptional analysis) of mixed intra-erythrocytic stages or mixed gametocyte stages were made on glass slides, fixed with 100% ice-cold methanol and 100% acetone at −20°C, washed three times with 1X PBS and blocked with 1X PBS/10% foetal bovine serum (FBS) for 4 hours. Slides were then incubated with either pre-absorbed control pre-immune serum or pre-absorbed anti-PF3D7_1363700 antibodies in 1X PBS/10% FBS (1:50) overnight at 4°C. Smears were washed three times with 1X PBS then incubated with control primary antibodies (1:200) (i.e., antimerozoite surface protein 1 (MSP1; MRA-94; MR4 ATCC) monoclonal antibodies for asexual stages or anti-Pfs230 (MRA-27; MR4 ATCC) monoclonal antibodies for gametocytes). Lastly, smears were washed with 1X PBS and double-labelled with TO-PRO-3 nuclear stain (1:50) (Molecular Probes) and secondary antibodies Alexa-488 and −586 (1:200) (Molecular Probes) for 2 hours at room temperature. After three washes, one drop of ProLong® Gold (Invitrogen) antifade reagent was added to the slide followed by a coverslip. Parasites were analysed using a Zeiss Laser Scanning Microscope (LSM) 510 META NLO two-photon point-scanning confocal microscope with a 100X oil immersion objective. An estimated 300 parasites were examined for each of the three biological replicates of mixed erythrocytic stages, while approximately 100 gametocytes (i.e., a mixture of I-V gametocyte stages) were examined for each of the two biological replicates.

Oocyst and salivary gland sporozoite slides were made as previously described [8,15]. Pre-absorbed pre-immune or anti-PF3D7_1363700 antibodies were diluted (1:25) in 1% goat serum plus 1% bovine serum albumin (BSA) and a volume of 40–100 μl were incubated on the slides overnight at 4°C. A 1:200 dilution of control primary antibody, anti-circumsporozoite protein (CS) MAb (MRA-183; MR4 ATCC) also incubated on the slides. Two biological replicates of oocyst sporozoites were performed and 150 oocyst sporozoites/replicate were counted and analysed for PF3D7_1363700 expression. At least two biological replicates of salivary gland sporozoites at days 14, 15, 16 and 20 PE were performed with 100–150 sporozoites/day/replicate observed. Additionally, one biological replicate of sporozoites at days 17, 18, 19, and 21 PE with a minimum of 20 sporozoites for each time point were observed.

### PF3D7_1363700-GFP construct design

*PF3D7_1363700* was PCR-amplified from a NF54 parasite strain genomic DNA template using forward primer and reverse primers, F: 5^′^-CCG*CTCGAG*GGAGCACATCGATGGGGAG-3^′^ and R: 5^′^*CCTAGG*ACTTGAGTCTAGACCTCTGGA-3^′^ (*XhoI* and *AvrII* restriction enzymes are italicized). The resulting product was cloned into the pPM2GT vector [16] to generate the plasmid designated as MpPM2GT. The schematic and standard protocol were previously described by Schlarman and colleagues, with the exception that the *PF3D7_1363700* product was ligated into the pPM2GT vector [8]. GFP expression was controlled by the endogenous promoter of *PF3D7_1363700* and parasites with the vector were selected using the drug-resistance cassette containing human dihydrofolate reductase (DHFR) sequence [8,17]. This MpPM2GT plasmid was sequenced to verify correct nucleotides and to ensure that the sequence was in frame, thereby allowing expression of GFP.

### Isolating clonal populations of PF3D7_1363700-GFP parasites and genotype analysis via PCR and Southern blot analysis

In order to transfect MpPM2GT into NF54 parasites, sorbitol-synchronized ring stage parasites at a 2-3% parasitaemia were electroporated with 50–100 μg of MpPM2GT plasmid DNA using low voltage conditions (BTX 600; 0.2 cm cuvette, 0.31 kV, 950 μF) [10,18]. Selection for parasites with plasmid integration into the genome (integrants) or parasites carrying episome was performed using 5 nM WR99210 (Jacobus Pharmaceutical Company Inc.) with three drug selection cycles (i.e., each WR99210 drug cycle = 4–6 weeks with drug and 3–4 weeks of drug removal) in order to enrich for integrants.

To assess the genotype of the parasite and detect if the parasites had successfully integrated the construct, a PCR analysis was performed using:

Wild-type control primers-F: 5^′^-CCGCTCGAGGGAGCACATCGATGGGGAG-3^′^ and R: 5^′^-CCTAGGACTTGAGTCTAGACCTCTGGA-3^′^; Episome control primers (hDHFR drug cassette) - F: 5^′^-ATGGTTGGTTCGCTAAACTGC-3^′^ and R: 5^′^-TTAATCATTCTTCTCATATACTTC-3^′^; Integration primers-F: 5^′^-TGATAAAAATGGACAAACAGGG-3^′^ and R: 5^′^-TCCGTATGTTGCATCACC-3^′^. PCR conditions were performed using the same conditions described earlier, however in a 20 μl reaction volume.

To further confirm the genotype, Southern blot analyses were done using 3–5 μg of DNA (i.e., either from *P. falciparum* NF54 genomic DNA, MpPM2GT plasmid DNA or MpPM2GT parasite culture DNA) that was digested with 20 units of *AleI* and *BamHI* restriction enzymes for 4 hours in a 37°C water bath. Digested DNA was separated on a 0.8% electrophoretic agarose gel and transferred to a nylon membrane (Osmonics). Membranes were UV crosslinked and prehybridized at 65°C in Church buffer (0.17% Phosphoric acid, 1% BSA, 1 mM EDTA, 7% SDS, 3.5% sodium phosphate dibasic) for 2 hours. After prehybridization, the membrane was hybridized with PF3D7_1363700-specific probes which were amplified using gene primer sets (F: 5^′^-CCGCTCGAGGGAGCACATCGATGGGGAG-3^′^ and R: 5^′^-CCTAGGACTTGAGTCTAGACCTCTGGA-3^′^) and digoxigenin (DIG; Roche Applied Science)-labelled.

Membrane and probe incubations were performed at 50°C in Church buffer overnight and washed twice with 2X SSC (3 M sodium chloride, 0.3 M sodium citrate, pH 7.0) containing 0.1% sodium dodecyl sulphate (SDS) at room temperature for 5 minutes and twice with 0.5X SSC containing 0.1% SDS at 65°C for 15 minutes. Detection of DNA was achieved using the DIG Kit (Roche Applied Science) according to the manufacturer’s instructions. CSPD disodium 3-(4-methoxyspiro {1,2-dioxetane-3,2^′^-(5^′^-chloro)tricyclo [3.3.1.1^3,7^decan}-4-yl)phenyl phosphate (Roche Applied Science) was used for chemiluminescence detection and membranes were exposed to X-ray film. Clonal populations were obtained by performing limiting dilutions [19]. Limiting dilutions were done by diluting parasites with culture medium to 0.5 and/or 0.25 parasites/well in two 96-well plates (i.e., one parasite every other well or every fourth well, respectively). Each well consisted of 200 μl of a 2.5% haematocrit and plates were placed in a gassed (3% O_2_, 3% CO_2_ and 94% N_2_) sealed Modular Incubator Chamber (Billups-Rothenberg Incorporated) at 37°C. To increase humidity, a small tissue culture plate with RPMI 1640 medium was placed inside the chamber. Chambers were gassed every second day, while culture medium was changed once a week with a fresh 0.4% haematocrit. On days 20–24, genomic DNA was isolated from each well to determine if clonal populations were present. Genomic DNA was isolated by removing 150 μl of the parasite culture and incubating the culture with 6% saponin (Sigma) for 5 minutes at room temperature. The 96-well plate containing this mixture was centrifuged at 2,650 × g for 15 minutes and the pellet was washed once with 1X PBS. Genomic DNA was extracted using 40 μl of down scale prep buffer (DSP; 1 M Tris-CL pH 8.0, 1 M KCL and 1 MgCl_2_) with an incubation of 50°C for 30 minutes and then 95°C for 10 minutes.

PCR was used to verify integration with the following conditions: 1 mM MgCl_2_, 0.2 mM dNTP, 1.25 U Taq polymerase (Promega), 0.5 mM of each primer, and 4 μl of gDNA isolated from the 96-well plate. PCR amplification was performed as follows: 1) initial denaturation at 95°C for 3 minutes; 2) denaturation at 94°C for 30 seconds; 3) annealing at 50°C for 45 seconds; 4) extension at 62°C for 3 minutes and 5) final extension at 62°C for 10 minutes. Each amplification cycle was repeated 34 times. Wells containing parasites as determined via PCR were expanded in 12-well plates at a 3% haematocrit in 2 ml of culture medium. Once the parasitaemia increased to approximately 2%, half the culture was cryopreserved in Glycerolyte 57 (Baxter) and stored in liquid nitrogen while the other half was expanded into a T25 flask (Corning Incorporated) at a 3% haematocrit in 10 ml of culture medium. Southern blot analysis as described previously was performed to confirm MpPM2GT clones.

### Fluorescence microscopy for GFP trafficking studies

Stable transgenic *P. falciparum* MpPM2GT parasite cultures were sorbitol-synchronized to allow specific intra-erythrocytic stage parasites to be analysed and enriched gametocyte stages were established as previously described. GFP trafficking studies were conducted as mentioned by Schlarman and colleagues [8]. Controls used for the GFP trafficking studies were *P. falciparum* NF54 wild-type parasites (negative control) and *P. falciparum* 3D7HT-GFP parasites (positive control) [9]. There were three independent biological replicates performed for each of the seven parasite stages analysed. For each biological replicate, the numbers for each stage analysed were: 50–100 trophozoites/schizonts/gametocytes, 2–5 zygotes, 5–15 oocysts with sporozoites, 2 haemolymph sporozoites [8,15], and ~20 salivary gland sporozoites for each day between day 14–20 PE.

## Results and discussion

### Bioinformatic sequence analyses of PF3D7_1363700

As a result of the *P. falciparum* genome sequencing project and large-scale microarray and proteomic studies, there are new opportunities to discover novel drug and vaccine candidates. Ideal targets to prevent malaria transmission are often surface antigens and secreted proteins involved in parasite invasion of the mosquito and human hosts [20-22]. In an effort to discover molecules involved in parasite invasion, the *P. falciparum* gene *PF3D7_1363700* (also known as *PfMAL13P1.319* and referred in the figures and figure legends as PfMal or PfM or M) was selected based on protein expression and signal peptide and localization predictions via *in silico* screening of PlasmoDB. PF3D7_1363700 is a 790-amino acid protein that encodes a 92.8-kDa protein including the signal peptide and a 89.1-kDa protein without the signal peptide. Signal peptide predictions, according to the SignalP and TargetP programs, indicated that PF3D7_1363700 has a signal peptide, and therefore should enter the secretory pathway. In support of this prediction, Softberry and PSORTII, two localization programs, predicted PF3D7_1363700 to be either a secreted or plasma membrane protein. Based on topology predictions, there is one transmembrane domain located within the signal peptide region, which would suggest that PF3D7_1363700 may be a secreted protein once the signal peptide is cleaved. The ScanProsite program identified many potential enzyme modification sites such as amidation, N-glycosylation and N-myristolation, which would suggest that the PF3D7_1363700 protein may be processed.

A putative indole-3-glycerol phosphate synthase (IGPS) domain was identified, according to analyses available on PlasmoDB [6]. IGPS is an enzyme that catalyses the fourth step in the tryptophan biosynthetic pathway (i.e., indole ring-closure reaction) where the substrate 1-(o-carboxyphenylamino)-1deoxyribulose-5^′^-phosphate (CdRP) is converted to indole 3-glycerol phosphate [23]. PF3D7_1363700 protein alignment with IGPS showed a 10.1% amino acid homology (specifically 5.0% identical amino acids) and no conserved catalytic residues of IGPS, specifically, were present. Based on these results, PF3D7_1363700 would not seem likely to function as a metabolic enzyme involved in tryptophan metabolism.

**Figure 1:**
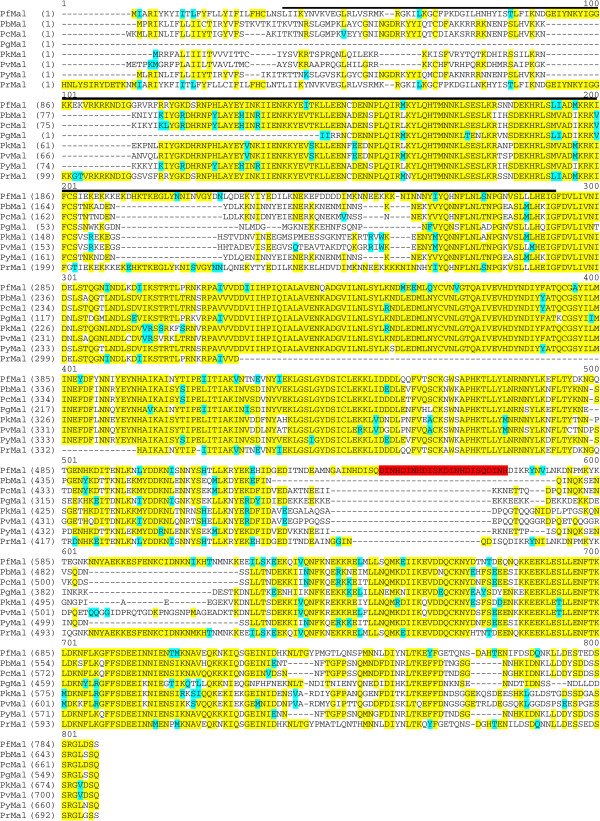
**Alignment of the PF3D7_1363700 (PfMal) protein sequence with its orthologues.** PF3D7_1363700 shares conserved regions with *P. berghei* (PbMal orf= PbANKA_113970 in PlasmoDB), *P. chabaudi* (PcMal orf= PCHAS_113920 in PlasmoDB), *P. gallinaceum* (PgMal orf= Pg_c000129384. Contig1-2-1079-2746), *P. knowlesi* (PkMal orf= PKH_110700 in Sanger Institute), *P. vivax* (PvMal= Pv115140)*, P. yoelii* (PyMal orf= chrPyl_00226-5-31074-29071 in PlasmoDB), and *P. reichenowi* (PrMal= reich142b09.plk, reich382d09.qlk, reich915f08.plk, reich838g12.plk in PlasmoDB). Identical residues are shaded in yellow while similar amino acids are shown in blue. The sequence used for antibody production is indicated by the black horizontal bar. The D-I-N-H repeat sequence is highlighted in red.

BLAST searches with PF3D7_1363700 in PlasmoDB showed significant identity and similarity with a protein in the human malaria parasites *Plasmodium vivax* (PVX_115140 in PlasmoDB; 60.8%) and *Plasmodium knowlesi* (PKH_110700 in PlasmoDB; 62.5%); avian malaria parasite *Plasmodium gallinaceum* (Pg_c000129384. Contig1-2-1079-2746 in PlasmoDB; 53%); rodent malaria parasites *Plasmodium berghei* (PbANKA_113970 n PlasmoDB; 60.3%), *Plasmodium chabaudi* (PCHAS_113920 in PlasmoDB; 63%) and *Plasmodium yoelii* (chrPyl_00226-5-31074-29071 in PlasmoDB; 61.6%) and the non-human primate parasite *Plasmodium reichenowi* (reich142b09.plk, reich382d09.qlk, reich915f08.plk, reich838g12.plk in PlasmoDB; 81%) (Figure 1). In addition, BLAST searches of EuPathDB demonstrated lower protein homology with other Apicomplexan proteins in *Toxoplasma gondii* (15.4%); *Neospora caninum* (14.6%); *Theileria annulata* (30%); and *Babesia bovis* (26%). There were no orthologues in other higher eukaryotic organisms suggesting that PF3D7_1363700 is unique to Apicomplexans. In addition, the alignment of the PF3D7_1363700 protein and its orthologues identified a four-amino acid repeat sequence of aspartic acid, isoleucine, asparagine, and histidine (D-I-N-H), which was found only in the PF3D7_1363700 orthologue in *P. falciparum*.

### *PF3D7_1363700* Transcript is present in intra-erythrocytic stage parasites, oocyst sporozoites and salivary gland sporozoites

**Figure 2:**
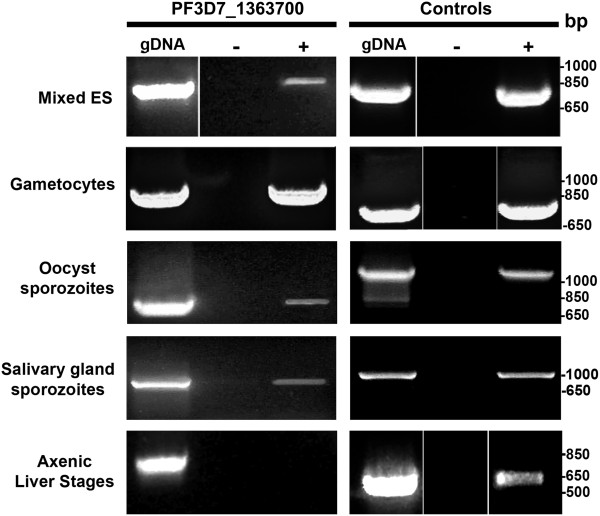
***PF3D7_1363700 *transcripts are present in various stages of *Plasmodium falciparum.****PF3D7_1363700* transcripts are present in asexual blood stages, gametocytes, oocyst sporozoites, and salivary gland sporozoites as determined by reverse-transcription PCR (RT-PCR). cDNA was produced from RNA isolated from mixed erythrocytic stages (ES), enriched gametocytes, salivary gland sporozoites, oocyst sporozoites, and axenic liver stages. PCR amplifications were performed using *PF3D7_1363700* specific primers and parasite control primers (+); *P. falciparum* merozoite surface protein 1 (MSP1) control primers for blood stage parasites, Pfs230 control primers for gametocytes, *P. falciparum* circumsporozoite (CS) primers for salivary gland and oocyst sporozoites and *P. falciparum* heat shock protein 70 (PfHSP70) primers for axenic liver stages. *Plasmodium falciparum* genomic DNA (gDNA) was used as a positive control while a no reverse transcriptase (−) reaction showed that there was no genomic DNA contamination. DNA size markers in base pairs (bp) are shown to the right of the figure.

Mixed asexual blood stages, enriched gametocyte cultures, day 8–10 PE oocyst sporozoites, day 14–17 PE salivary gland sporozoites and axenic liver stages for *PF3D7_1363700* were analysed for transcript expression. *PF3D7_1363700* transcripts were present throughout all the tested stages except axenic liver stages (Figure 2). Axenic liver stage parasites produced in the absence of liver cells were analysed since there was no success producing liver stages when using primary human hepatocytes. A *PfHsp70* gene control was used to show the presence of liver stage parasites, which were produced by the axenic cultures. Stage-specific controls were used as a positive control to demonstrate presence of parasites in all experiments.

Previous transcript expression profile data for *P. falciparum* were reported by Le Roch and colleagues who used a high density oligonucleotide array to assess the transcriptome of mosquito salivary gland sporozoite-, ring-, trophozoite-, schizont-, merozoite- and gametocyte-stage parasites [24]. Results from their whole genome wide study showed transcript presence throughout all the stages observed and complemented the higher accuracy RT-PCR data generated in this single gene study, for mixed erythrocytic stages, gametocytes and salivary gland sporozoites. In addition, researchers analysed transcript expression of activated sporozoites that were ready to invade hepatocytes by incubating sporozoites for one hour at 37°C with hepatocytes [25] and results showed *PF3D7_1363700* transcript to be upregulated in comparison to pre-activated salivary gland sporozoites. This result would suggest that *PF3D7_1363700* along with other upregulated genes may prepare or help activate the sporozoite for successful infection of the host tissue or be necessary for early stages of hepatocyte infection. *PF3D7_1363700* transcript analysis in the oocyst sporozoites and axenic liver stages has not been previously reported in the literature and demonstrates new knowledge of transcript expression in these stages. Overall, a diverse transcript expression profile of *PF3D7_1363700* demonstrated transcript presence in many parasite stages.

### Analysis of PF3D7_1363700 protein presence in the intra-erythrocytic stages

**Figure 3:**
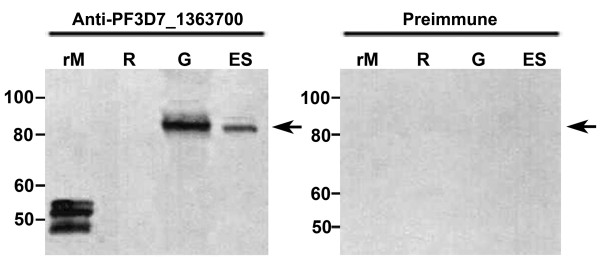
**PF3D7_1363700 protein is present during intra-erythrocytic stages via Western blot analysis.** PF3D7_1363700 protein was recognized by anti-PF3D7_1363700 antibodies in the *P. falciparum* asynchronous asexual blood stages (ES) and enriched gametocyte (G) lysates via Western blot analysis (as denoted by the arrow). The pre-immune antibody did not detect this protein. The recombinant PF3D7_1363700-A protein (rM) was used as a positive control and non-infected RBCs (R) were used as a negative control. Molecular weight markers in kilodaltons are shown to the left of each figure. Three independent biological replicates were performed and representative images are shown.

To assess the protein expression profile of PF3D7_1363700 during the intra-erythrocytic stages, *P. falciparum* asynchronous intra-erythrocytic stage lysates were immunoblotted with anti-PF3D7_1363700 antibodies. By Western blot analysis, PF3D7_1363700 was detected in mixed *P. falciparum* intra-erythrocytic parasites and enriched gametocyte lysates, suggesting that the PF3D7_1363700 protein is present during the erythrocytic stages (Figure 3). The band representing PF3D7_1363700 appeared near the calculated molecular mass of 92.8 kDa with the signal peptide and 89.1 kDa without its signal peptide. Anti-PF3D7_1363700 antibodies did not react against non-infected red blood cells (RBCs), while pre-immune control serum exhibited no cross-reacting antibodies against intra-erythrocytic lysates, recombinant PF3D7_1363700-A, or RBCs.

**Figure 4:**
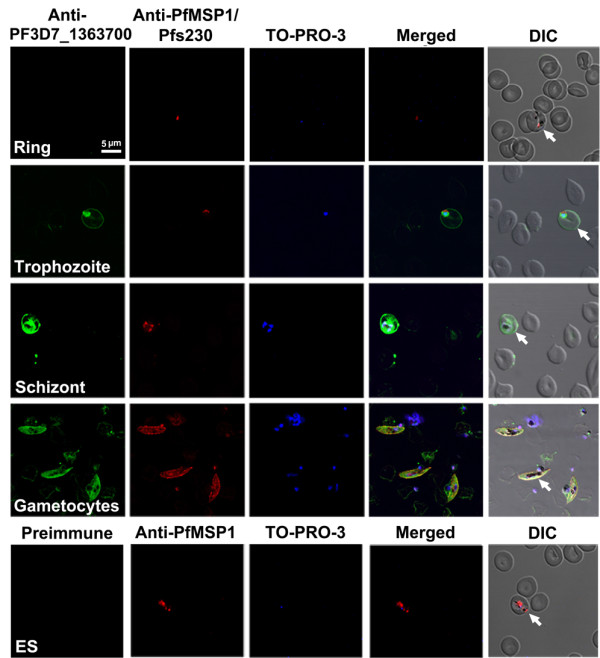
**PF3D7_1363700 protein appears to be present during late intra-erythrocytic stages as shown by immunolocalization studies using confocal microscopy.** Parasite-infected blood smears were triple-labelled with anti-PF3D7_1363700 polyclonal antibody (anti-PF3D7_1363700), either anti-merozoite surface protein 1 (anti-PfMSP1) monoclonal antibody for asexual stages or anti-Pfs230 monoclonal antibody for gametocytes (used as positive controls), and a nuclear stain, TO-PRO-3. Triplicate biological replicates of parasite-infected blood smears consistently show PF3D7_1363700 expression during the trophozoite, schizont and gametocyte stages. Merged images with Differential Interference Contrast (DIC) demonstrate protein co-localization with known surface proteins as shown by the yellow colour. Approximately 100–300 stage-specific parasites/replicate were observed. The bottom panel of figures show the control pre-immune serum with minimal cross-reactivity in intra-erythrocytic stage parasites (ES). Arrows denote the presence of parasites and representative images are shown with a scale bar, 5 μm.

To ascertain PF3D7_1363700 protein presence on a single-cell level, immunofluorescent assays were performed using anti-PF3D7_1363700 antibodies with blood smears of mixed *P. falciparum* intra-erythrocytic stages (i.e., rings, trophozoites, schizonts and gametocytes) and a mixture of stage I-V female and male gametocytes. The PF3D7_1363700 protein was shown to be present during the trophozoite, schizont and gametocyte stages (Figure 4). Triplicate biological replicates of approximately 100–300 stage-specific parasites/replicate consistently showed PF3D7_1363700 expression with an average percentage of PF3D7_1363700-expressing parasites of 100% for trophozoite and schizont stages and 57% for gametocyte stages mixed with stage I-V gametocytes. There was no detection of PF3D7_1363700 protein during the ring stages, suggesting that PF3D7_1363700 expression is time and stage-specific. Using PfMSP-1 antibodies and Pfs230 control antibodies, PF3D7_1363700 co-localized with the surface protein PfMSP-1, albeit at a low resolution. Further high-resolution studies are required in order to determine if PF3D7_1363700 is a surface protein or if it specifically localizes to other areas of the parasite.

**Figure 5:**
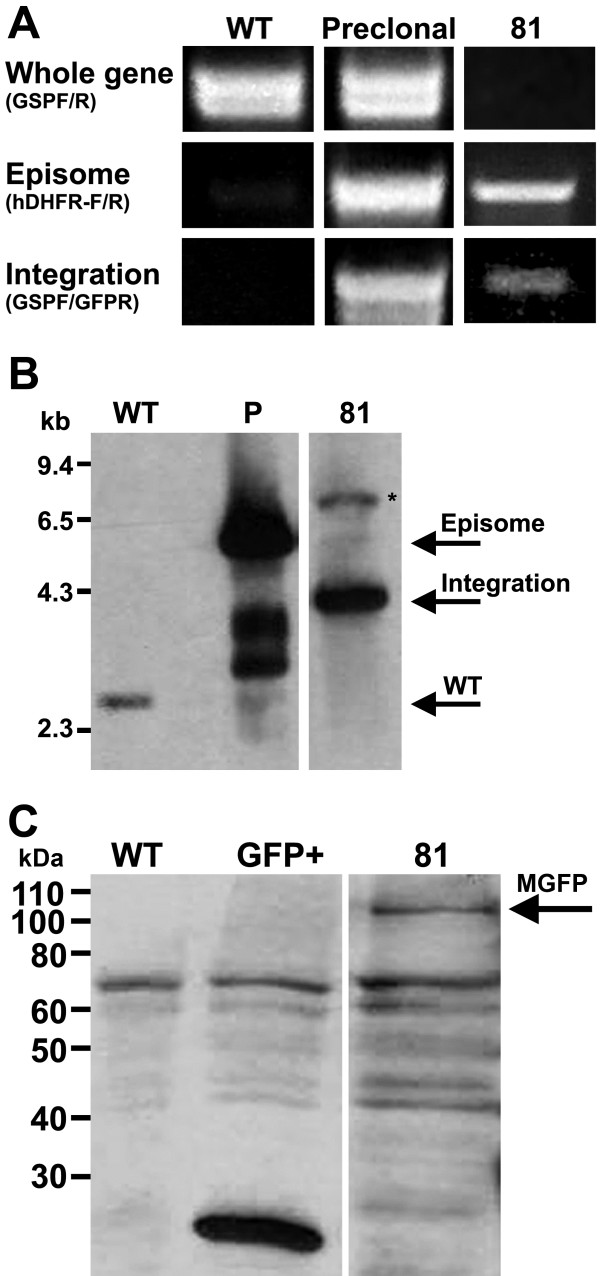
**A-C. Generation of PF3D7_1363700-GFP clonal populations that appear to express PF3D7_1363700-GFP during the intra-erythrocytic stages.** A stable integration event into the *PF3D7_1363700* chromosomal locus was detected via genotype analyses. (**A**) For PCR analysis, a genomic DNA template from wild-type parasites (WT) was used as a control and MpPM2GT transfected culture DNA before limiting dilution (Preclonal) was used to demonstrate the presence of integrants and wild-type parasites carrying the episome. Clonal population MpPM2GT-81 (81) was the experimental template. PCR primer combinations listed to the left of the figure were used to detect: 1) wild-type parasites: gene specific forward and reverse (GSPF/R); 2) episomes: hDHFR F/R and 3) parasites with integration: gene specific forward (GSPF) and GFP reverse (GFPR). The faint band that represents the detection of episome in wild-type control DNA was due to primers binding to DNA at a temperature lower than the ideal annealing temperature. At ideal annealing temperatures, this band is not present. (**B**) For Southern blot analysis, integration was detected by the presence of a 4.2-kb band while wild-type parasites with episome were represented by 2.7-kb and 5.1-kb bands. The asterisk denotes an unknown band, which could be due to plasmid rearrangement after transfection. Genomic DNA from wild-type *P. falciparum* NF54 parasites (WT) and transfected episome (P) were used as controls in the Southern blot experiments and a digoxigenin-labelled *PF3D7_1363700* DNA probe was used for detection. (**C**) PF3D7_1363700-GFP fusion protein (MGFP) from clonal population 81 appears to be present using anti-GFP antibodies for detection as denoted by the arrow. Wild-type (WT) and 3D7HT-GFP (GFP+) parasites were used as negative and positive controls, respectively, in the Western blot.

**Figure 6:**
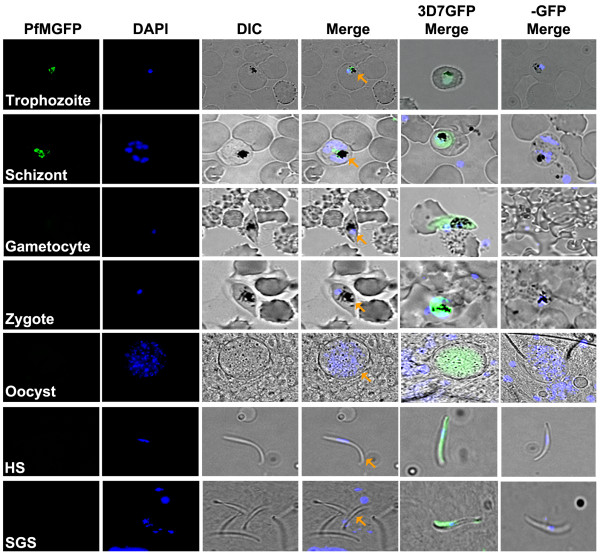
**PF3D7_1363700-GFP expression in various parasite stages.** The PF3D7_1363700-GFP fusion protein of clonal population 81 (PfMGFP) was expressed only during the late asexual blood stages such as the trophozoites and schizonts. No expression was observed during the ring stage (data not shown), gametocyte stage and mosquito parasite stages (i.e., zygote, oocyst sporozoite, haemolymph sporozoite (HS), and salivary gland sporozoite (SGS). Expression of the fusion protein was under the control of the PF3D7_1363700 promoter. DAPI was used for parasite nuclei staining. Orange arrowheads denote the presence of parasites in the merged images. 3D7GFP merge images depict constitutively expressing parasites that were used as a positive control and –GFP merge are images of wild-type parasites that were used as a negative control. Fluorescent images shown are representative of PF3D7_1363700 expression during that specific stage and three biological replicates were performed for each stage observed.

In addition to IFA, a GFP-trafficking approach using the endogenous *PF3D7_1363700* promoter was performed to investigate the presence of PF3D7_1363700 protein in the mosquito and human host parasite stages. The 3^′^-end of *PF3D7_1363700* was cloned in-frame with GFP in the pPM2GT vector [16], yielding MpPM2GT. Following drug selection for mutant parasites and limiting dilutions, a clonal population (clone-81) was isolated and showed the presence of pPM2GT plasmid integration via PCR and Southern blot analysis (Figure 5A-B). To assess if MpPM2GT clone-81 parasites express PF3D7_1363700-GFP protein during the intra-erythrocytic stages, anti-GFP antibodies were used. Expression of the PF3D7_1363700-GFP fusion protein was detected at ~110 kDa (Figure 5C). In addition, MpPM2GT-Clone 81 was analysed on a single-celled level and similar to the IFA results, green fluorescence was detected in trophozoites and schizonts while ring stages displayed minimal to no expression (Figure 6). On the other hand, gametocyte stages, similar in number and specific stages observed in IFA had no detection of GFP fluorescence. This result could be attributed to GFP expression having a lesser detection signal than immunocytochemistry using Alexa-conjugated secondary antibodies that gives a stronger signal [26] and/or the possibility of PF3D7_1363700-GFP protein being misfolded or processed differently than wild-type PF3D7_1363700 protein [27]. These results were confirmed by three independent biological replicates of 50–100 trophozoites/schizonts/gametocytes per replicate in order to support the validity and reproducibility of these results. Controls used for the GFP trafficking studies were the negative control, *P. falciparum* NF54 wild-type parasites, and as a positive control *P. falciparum* 3D7HT-GFP which is a parasite line that constitutively expresses the GFP protein in all parasite stages [9].

It has been reported in the literature that there is no detection of the PF3D7_1363700 protein during the intra-erythrocytic stages using high-accuracy mass spectrometry [28,29]. While Lasonder and colleagues analysed trophozoites, schizonts, gametocytes, and gametes and Florens and colleagues observed merozoites, trophozoites and gametocytes, both sets of authors did not report the presence of the PF3D7_1363700 protein during these observed stages. Lasonder and colleagues reported that PfEMP1, a known asexual stage specific protein, surprisingly did not exhibit high-scoring peptides, which they stated could be due to poor extraction of the proteins [28]. Lasonder and colleagues also rejected low scoring proteins, thereby suggesting that PF3D7_1363700 may not have made the cut-off score and thus, was not considered significant. Another explanation for the differences in results is the type of technique used to assess protein expression. The previous reported studies are based on a global profile, large-scale mass spectrometry approach while the IFA and GFP studies were performed on a specific, single-cell level and Western blot analysis utilizes detection kits that are sensitive enough to detect extremely low levels of protein. In addition, both sets of authors synchronized the asexual blood stages and collected these parasites at specific time points, which may have prevented the analysis of proteins expressed transiently before the collection time points, and therefore they did not detect the PF3D7_1363700 protein.

### Analysis of PF3D7_1363700 protein expression in the mosquito parasite stages

PF3D7_1363700 protein expression during the mosquito parasite stages, which include zygotes, oocyst sporozoites, haemolymph sporozoites and salivary gland sporozoites, was also investigated via IFA and GFP trafficking studies. Anti-PF3D7_1363700 antibodies did not detect PF3D7_1363700 protein in day 10 PE oocyst sporozoites and salivary gland sporozoites of days 14, 15, 16, and 20 PE. To confirm results, two biological replicates of 100–150 oocyst and salivary gland sporozoites were observed using antibodies to the circumsporozoite (CS) protein and pre-immune serum as a positive and negative control, respectively. Although one IFA biological replicate did detect PF3D7_1363700 protein, this result was not reproducible. The possibility of the PF3D7_1363700 protein being specifically expressed at different time points also was addressed by observing sporozoites every day between days 14 and 21 PE. However, there continued to be no detectable protein.

In addition to the IFA results, GFP-trafficking studies examined these same sporozoite stages as well as zygotes and haemolymph sporozoites (Figure 6). Three independent biological replicates of the GFP studies also supported that PF3D7_1363700 was undetectable during these parasite stages in the mosquito. 3D7HT-GFP parasites were used as a positive control with GFP-expressing parasites observed for all tested stages. Thus, PF3D7_1363700 was either expressed in undetectable amounts or not expressed during these mosquito parasite stages. Previous high-throughput mass spectrometry by Florens and colleagues identified PF3D7_1363700 present in day 14PE salivary gland sporozoites [29]; however the authors detected a low specrum number of 1 for PF3D7_1363700 in comparison to the significantly-expressed sporozoite surface protein CS, which had a spectra number of 10. This result along with IFA and GFP-trafficking data reported herein would suggest that PF3D7_1363700 is likely expressed at very low levels. In addition, there was only one peptide match barely above the 95% confidence level in a global proteomic expression analysis, suggesting that PF3D7_1363700 protein presence during the salivary gland sporozoite stage is dependent upon the identification of this single correct peptide sequence in a large mass of other proteins and any misidentification in the peptide sequence would result in no detection of PF3D7_1363700. Also, a cross-correlation score of 1.812 was exactly at the cut-off score for detectable proteins, which also supports the low level detection of PF3D7_1363700. Predictions of PF3D7_1363700 protein being a secreted protein could also be a reason why the protein was difficult to detect, especially if the protein was secreted from the sporozoite and released in the mosquito.

### Multiple attempts at isolating *PF3D7_1363700*-disrupted mutant parasites were not successful

To assess the role of PF3D7_1363700 during the late intra-erythrocytic stages, a classical single-crossover gene disruption of *PF3D7_1363700* was performed. In order to disrupt *PF3D7_1363700*, 441 or 710 bp of the 5^′^-end of *PF3D7_1363700* was cloned into pCAM-BSD [30] (i.e., designated M441pCAM and M710pCAM), which utilizes the blasticidin S deaminase (BSD) drug cassette and then transfected into *P. falciparum* NF54 parasites. The genotype of drug-resistant parasites was analysed after each round of drug selection via PCR analysis and it was discovered that M441pCAM and M710pCAM parasites displayed evidence of an integration event; however wild-type parasites with the episome were also present.

After this observation, multiple attempts were made to isolate a clonal population of the *PF3D7_1363700*-disrupted parasites via limiting dilutions. However after PCR analysis, each potential clonal population always had wild-type parasites with episome present. For M441pCAM genotype analyses, seven populations out of 784 wells had both parasites with integration and wild-type parasites with episome and M710pCAM genotype analyses resulted in four populations out of 392 wells that were also mixed with integrants and wild-type parasites with episomes. Therefore, no population with only parasites with integration was detected. The inability to separate the integrants from wild-type parasites with the episome could be the result of wild-type parasites with the replicating episome outgrowing the integrants [31,32]. Hence, the *PF3D7_1363700* gene may be needed for parasite growth or survival. Some *Plasmodium* intra-erythrocytic stage proteins that are essential for parasite survival, such as apical membrane associated protein (AMA-1), falcipain-3 (FP3), calpain, and merozoite surface protein (MSP-1_19_) genes, have resulted in failed disruption and deletion attempts due to the lethal nature of the gene manipulation [33-37]. Therefore, the failed attempts to obtain a *PF3D7_1363700*-disrupted clonal population would support a potential role of PF3D7_1363700 that is critical to the parasite.

## Conclusions

Identifying and characterizing gene candidates involved in host-parasite interactions, development, and metabolism throughout the entire life cycle of *P. falciparum* is a major asset to understanding the overall biology of malaria parasites and discovering interesting candidates for vaccine/drug intervention. This research addresses a once uncharacterized *Plasmodium* gene, *PF3D7_1363700*, which was identified by data mining of PlasmoDB. PF3D7_1363700 has an N-terminal signal sequence, is predicted to be a secreted protein, and is a well-conserved protein in many *Plasmodium* species. The transcript expression profile of *PF3D7_1363700* obtained via RT-PCR and complemented by DNA micro-array studies described in the literature, demonstrated transcript presence in oocyst and salivary gland sporozoites of the mosquito host and intra-erythrocytic stages of the human host [24,25]. Although initial screening via PlasmoDB characterized PF3D7_1363700 as a sporozoite protein, the current study demonstrates PF3D7_1363700 protein expression only during the late intra-erythrocytic stages. Functional studies were attempted to identify a specific role of PF3D7_1363700; however it was not possible to isolate a *PF3D7_1363700*-disrupted clonal population because wild-type parasites with the episome were always present. Since some previously studied intra-erythrocytic stage proteins cannot be disrupted or deleted, it has led to the possibility that these genes may be essential for parasite growth or survival [33-37]. Therefore, it is possible that PF3D7_1363700 may participate in a mechanism essential for parasite survival.

## Competing interests

The authors declare that they have no competing interests.

## Authors’ contributions

RR designed and carried out experiments, analysed data and wrote the final manuscript. MS, AL, and MK assisted in experimental design and manuscript edits. RO assisted with providing experimental reagents. BTB evaluated and helped coordinate experimental designs, analysed data, critically revised the manuscript and gave the final approval for its publication. All authors read and approved the final manuscript.
